# Ghrelin Immunoreactive Cell Amounts in the Abomasum in 4-Month-Old Calves by Feeding Different Amounts of Prebiotics and New Synbiotics

**DOI:** 10.1155/2021/5542372

**Published:** 2021-09-20

**Authors:** Astra Arne, Aija Ilgaza, Liga Astra Kalnina

**Affiliations:** ^1^Latvia University of Life Sciences and Technologies, Faculty of Veterinary Medicine, K. Helmaņa Street 8, Jelgava 3004, Latvia; ^2^St. John Fisher College, Biology Department, 3690 East Ave, Rochester, New York 14618, USA

## Abstract

The study aim was to determine prebiotic (inulin) and new synbiotic (inulin and *Enterococcus faecium)* varied dosage effects, during food breakdown-abomasum immunoreactive (IR) cell amount and cold carcass weight. Ghrelin is synthesized in the fundus region of the stomach. In the gastrointestinal system, ghrelin affects multiple functions, including secretion of gastric acid, gastric motility, and pancreatic protein output. The study consisted of 49 Holstein male calves (23 ± 5 days old, 50 ± 5 kg). Control and experimental groups were differentiated only with the additive amount added to the morning food source. Three prebiotic groups were fed Jerusalem artichoke flour (inulin content increased by 50%) in three amounts: 6 g (lowest) PreG_6_, 12 g (medium) PreG_12_, and 24 g (highest) PreG_24_. Three synbiotic groups were added 0.25 g of prebiotic *Enterococcus faecium* (2^*∗*^109 CFU/g) to the respective prebiotic, obtaining a new synbiotic (SynG_6_, SynG_12_, and SynG_24_). Calves were slaughtered after 56 days to obtain abomasum samples for ghrelin IR cell examination, and carcass weight was determined. It shows that ghrelin IR cell count in the abomasum was (*p* < 0.05) reduced in 6g and 12g inulin dosage, but carcass weight was significantly (*p* < 0.05) higher for PreG_12_ and PreG_24_ (*p* < 0.05) and then for CoG (CoG 42.6 kg; PreG_12_ 51.4 kg; and PreG_24_ 54.0 kg) and (*p* < 0.05) for SynG_12_ and SynG_24_ (SynG_12_ 52.3 kg and SynG_24_ 49.6 kg), which indicates longer satiety and more wholesome breakdown of the food uptake. It was concluded that ghrelin IR cells in 12-week-old calves are more abundant in the fundus region. Medium- and high-dosage prebiotic inulin feeding to the calves improves overall food digestion, allowing for longer satiety and higher cold carcass weight without increasing food amount. Adding synbiotic 0.25 g *Enterococcus faecium* (2^*∗*^10^9^ CFU/g (Protexin, UK)) to inulin (produced in Latvia LTD „Herbe”) does not improve the results of this prebiotic.

## 1. Introduction

The search for methods in agriculture animal farming continues to find methods to increase farm animal growth, development, and productivity, as well as disease prevention without the use of antibiotics [1]. It is important for cattle growing, when calves transition from milk being their main source of protein digestion in the abomasum to digestion of roughage and concentrates in the foregut. During the transition to a different mode of nutrient acquisition, food digestion and weight increase can significantly decrease and overall health can worsen.

The addition of prebiotics and probiotics or a combination of both, also known as synbiotics has been recognized as one of the most progressive methods for calves. Prebiotics contain oligosaccharides that are indigestible by enzymes that can ferment digestive tract bacteria by obtaining energy, modeling their growth and activity that improves gastrointestinal tract function, immune system, and health as a whole [2–4]. Probiotics are composed of viable microorganisms needed for digestive tract function that decrease pathogens in the digestive tract, facilitate weight increase, and optimize immune system function by leaving an overall positive effect of the whole organism [5, 6]. Prebiotics are used together with probiotics as a synbiotic to increase the positive effects [1, 7]. There is a search for a synbiotic that provides additional advantages to animal growth, food metabolism, and health ratings in contrast to addition to probiotics or prebiotics. Moreover, synbiotics are being made that can decrease GHG emission and increase food uptake digestion and other nutrients such a vitamin, microelement, and protein availability [5, 6]. With increased food digestion and absorption in animals with identical nutrient uptake, hunger decreases that lowers ghrelin immunoreactive (IR) cell activity.

Ghrelin was first described as a 28 amino acid intestinal peptide and growth hormone secretion receptor 1A endogen ligand in 1999 [8]. Ghrelin is a potential hunger stimulant that is primarily synthesized by the parietal cells in the stomach (ruminant abomasum), as well as in the epithelial cells of the large and small intestine [9–11]. This peptide is part of the energy metabolism, food uptake, and control of growth hormone secretion and plays an important role as a bone and cartilage homeostasis mediator as well as cell proliferation modeling [8, 12–14].

Its production in monogastric animals decreases rapidly after feeding and remains at low levels as long as the animal is not hungry. Ghrelin IR cells activate once the stomach is empty, which stimulates stomach motility and hydrochloric acid secretion, which stimulates hunger and the search for food. Cytoplasmic ghrelin secreted from stomach ghrelin cells promotes an increase in plasma ghrelin concentration. Ghrelin is known as a hunger signal from peripheral tissue, which indicates ghrelin IR cells are affected by peripheral cell metabolism [12, 15].

Due to the continuous flow of food from the foregut, ruminants have a different circadian rhythm than monogastric animals. There is a need for studies focusing on the role of ghrelin in a grown cattle organism. Before the complete transition from milk to roughage and concentrate breakdown in the foregut, ghrelin secretion in abomasum tissue could be dependent on the same laws as in monogastric animals. If calves are fed with Jerusalem artichoke flour containing prebiotic inulin (∼50%) and its combination with *Enterococcus faecium*, then food digestion improves by reducing the number of hunger hormone ghrelin IR cells in the abomasum.

This study aimed to determine ghrelin immunoreactive cell activity from 4-month-old calf abomasum as well as determine how prebiotics (inulin) and synbiotics (inulin and *E. faecium*) in different dosages impact the abomasum IR cell activity using cold carcass weight.

## 2. Materials and Methods

### 2.1. Experimental Design, Dietary Treatments, and Animal Management

The study was conducted at the LLU Veterinary Medicine Department, Preclinical institute. The study was based on 49 Holstein male calves that, at the start of the study, were 23 ± 5 days old, with an average weight of 50 ± 5 kg. Calves were assigned to 7 different groups, with 7 animals in each that differed in food uptake. All calves were fed twice a day with 4 l of milk with or without food additives. All animals had *ad libitum* access to hay and water. The control group (CoG) were only fed with whole milk. Prebiotic group calves were fed milk with Jerusalem artichoke (produced by “LTD Herbe”) additive which had an inulin concentration of 50% because usually, Jerusalem artichoke contains a 15–20% inulin concentration on its own [5, 6, 16]. Prebiotic group calves were fed milk with the following Jerusalem artichoke additive to milk: 6 g Jerusalem artichoke powder (3 g inulin; denoted PreG_6_); 12 g Jerusalem artichoke powder (6 g inulin; denoted PreG_12_); and 24 g Jerusalem artichoke powder (12 g inulin; denoted PreG_24_). Synbiotic group calves were fed milk with the Jerusalem artichoke powder and probiotic: 6 g Jerusalem artichoke powder (3 g inulin + 0.25 g *Enterococcus faecium* (2^*∗*^10^9^ CFU/g), denoted SynG_6_); 12 g Jerusalem artichoke powder (6 g inulin + 0.25 g *Enterococcus faecium* (2^*∗*^10^9^ CFU/g), denoted SynG_12_); and 24 g Jerusalem artichoke powder (12 g inulin + 0.25 g *Enterococcus faecium* (2^*∗*^10^9^ CFU/g), denoted SynG_24_). After the experiments' second week, calves were offered concentrates after being fed with milk. Concentrates were made on site and did not contain any growth stimulants or antibiotics.

### 2.2. Sample Collection and Weighing

After 56 days, animals were slaughtered (average of 12 weeks of age). After slaughter, histological samples from each calf (*n* = 49) abomasum's two parts *pars pylorica* and *fundus abomasum* were collected, which were rinsed with 0.9% NaCl solution and placed in 100 mL of 10% of formalin. Tissue cultures were fixed in 10% formalin solution for at least 48 hours. Carcasses were cooled, and the official weight recorded was fixed on the verified slaughterhouse scale.

### 2.3. Immunohistochemistry Analysis

Ghrelin immunoreactive (IR) cell detection was performed using immunohistochemistry staining methods. IR cell staining was performed with the streptavidin-biotin complex (Dako REAL™ EnVision™ Detection System, Peroxidase/DAB+, Rabbit/Mouse). Tissue samples were placed on microscope slides with silane coating (Histo Bond®) and dried for 12 hours in the thermostat at 37°C. Tissue samples underwent deparaffinization in xylitol and dehydration with an ethanol concentration-reducing column; samples were placed in 65°C buffer solution at pH 9 (Target Retrieval solution, pH 9, Dako). After heating, the samples were cooled and applied to endogen peroxidase blocking reagent (Dako Endogenus enzyme block) for 5 minutes. Rat-mouse polyclonal antibodies (Phoenix Pharma. Inc.H- 031-31) were used as primary antibodies diluted 1 : 500. To determine immunoreactive cell and primary antibody reaction, the samples were stained applying DAB + complex (Dako REAL™ EnVision™ Detection System). To avoid artefacts and to increase contrast, tissues were stained with hematoxylin. There was a negative control added without primary antibodies. Immunoreactive cell quantitative compositions were evaluated in every sample in 10 fields of vision to determine the immunoreactive cell number in 1 mm^2^. Samples were examined under 40x magnification using a light microscope Leica DM 500B by using Image Pro Plus 6.1. program. A total of 98 samples were examined and evaluated.

### 2.4. Statistical Analysis

To describe the results and to determine if there is a statistical difference between the two groups, the function average (AVERAGE) and standard deviation (STDEV), as well as the *t*-test (T.TEST), were performed to compare two groups. All statistical analysis was reported significant for tests with *p* < 0.05. All parameters were analyzed, and statistical analysis was conducted using Excel 2013 and SPSS Statistics-22 versions.

## 3. Results

**Figure 1:**
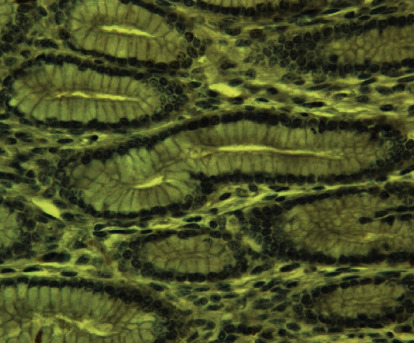
*Abomasum pars pylorica* in 4-month-old calf (PreG_24_), no ghrelin IR cells observed (400x, DAB + hematoxylin).

**Figure 2:**
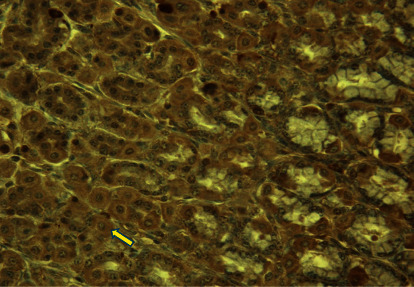
Ghrelin IR cells (arrow) in 4-month-old calf (CoG), *abomasum fundus* region (400x, DAB + hematoxylin).

In the *Abomasum pars pylorica* gland section (Figure 1) PreG_24_ group calf sample, no ghrelin IR cells were observed. In CoG, *pars fundalis* (Figure 2) IR cells were observed, and the arrow points to the reaction in the gland tissue cytoplasm as brown-colored granules.

Ghrelin immunoreactive (IR) cells are localized in *the abomasum* muscle gland cell cytoplasm in *the pylorus* and *fundus* sections. In the *fundus* region, more are seen in gland cell apical ends, but in the *pylorus* region, more are seen in gland cell nuclei and perinuclei. Ghrelin IR cells stain brown and are mostly observed to be round, oval, and, in some cases, square, stained with DAB + ghrelin granules colored brown (Figure 2). The negative control did not show positive staining results.

Examining ghrelin IR cell numbers in all experimental groups, they are significantly (*p* < 0.09) more abundant in *the abomasum fundus* region than in *the pylorus* region (Table 1).

Control group animals in contrast with all prebiotic and synbiotic group animals were observed to have significantly (*p* < 0.01) more IR cells in *the abomasum pars* region (see Table 1); PreG_6_ group animals were also found to have a high cell amount, and they are significantly (*p* < 0.05) more than PreG_12_ and PreG_24_. The SynG_6_ group`s ghrelin IR cell amount was significantly (*p* < 0.05) higher than that of SynG_12_ and SynG_24_ groups. It can be concluded that inulin addition to food of 6 g and 12 g significantly decreases ghrelin IR cell numbers in *the abomasum pars pylorica* region.

Analyzing *the abomasum fundus* region (see Figure 2) results, it can be seen that ghrelin and IR cells in CoG are significantly (*p* < 0.01) more than for PreG_12_, PreG_24_, SynG_6_, SynG_12_, and SynG_24_ group animals. In the SynG_6_ group, they are significantly (*p* < 0.01) more than SynG_12_ in SynG_24_. Group SynG_12_ ghrelin IR cell number was significantly higher (*p* < 0.01) than that of PreG_24_ group (5 ± 3.45 and 2 ± 0.97).

CoG animals reported the lowest cold carcass weight which was significantly (*p* < 0.01) lower than that of PreG_12;24_ and SynG_12_, as well as significantly (*p* < 0.05) lower than that of the SynG_6;24_ group. Between CoG and PreG_6_ group animals, there were no significant differences in cold carcass weight. The highest cold carcass weights were reported for calves that were fed the medium and higher dose of inulin.

## 4. Discussion

In this study, we would see that ghrelin immunoreactive cells can be found in the abomasum of 12-week-old calves. This supports other author findings that these cells can be found in as young as 2-week-old calves as well as old as 5-year-old or older cows' abomasum [9, 10]. Their numbers significantly differed between the two abomasum regions, *abomasum fundus* and *pars pylorica*. Our study control group and experimental group calves, ignoring food additive to milk amount, reported significant (*p* < 0.05) ghrelin immunoreactive cell numbers in *abomasum fundus* than the *pars pylorica* region (see Table 2). Other author studies support these findings of more IR cells in *the abomasum fundus* than *the pars pylorica* region [8, 14]. Their amount in the stomach after birth significantly increased in the first 5 postnatal development weeks. For calves at 12 weeks, their abomasum gland cells have developed enough to respond to satiety with secretion of ghrelin [13].

Ghrelin develops in the stomach and other digestive tract region mucus of hungry animals. Research with rats and humans showed that ghrelin presence in peripheral blood circulation in rats after gastrectomy reduces by 80% and by 65% in humans [17, 18], which points to the presence of ghrelin in peripheral blood circulation and the feeling of hunger is directly impacted by ghrelin secreting cells in the stomach. To determine the impact of different doses of prebiotics (inulin) and synbiotics (inulin and 0.25 g *Enterococcus faecium* (2^*∗*^10^9^ CFU/g)) on satiety in calves, we focused on ghrelin immunoreactive cell changes in the abomasum. It allows us to understand and determine the impact of additives on food breakdown because an empty stomach signals ghrelin production which will travel to the peripheral blood circulation. Rise in ghrelin levels signals from the peripheral to central nervous system and signals feeling hungry. Search for food, increased stomach acid secretion, and stomach motility were stimulated as well as *n.vagus* activity. The role of ghrelin in the releasing of growth hormone from hypophysis is to stimulate the search for food and if enough nutrients are present leading to increase in weight as well [8, 19–21].

Despite the overwhelming evidence that ghrelin IR cells are found in the stomach of ruminants [9–11], no studies were found on the impact of synbiotic and prebiotic additives to food regarding calf abomasum IR cell activity. This study provides unique findings on inulin (prebiotic) and its combination to 0.25 g of *Enterococcus faecium* (2^*∗*^10^9^ CFU/g) (synbiotic) uptake on IR cell amount in the calf abomasum region, ghrelin granule distribution, and amounts.

For calves which were fed the lowest dose of prebiotics, IR cells were observed at a relatively high amount. In [20], it was observed that a sufficient dose of prebiotics reduces ghrelin secretion, which in turn helps regulate calf weight, which was observed in our study as well. Medium- and high-dose prebiotic group calves with equal food uptake as the control group were found to have low numbers of ghrelin of IR cells in the abomasum than the control and low-prebiotic-dose calf groups. This shows that inulin in sufficient amounts (at least 6 g/per day) in 12-week-old calves significantly increases food uptake and breakdown, therefore decreasing the feeling of hunger. However, 0.25 g of prebiotic *Enterococcus faecium* (2^*∗*^10^9^ CFU/g) addition to the medium and the highest dose (6 and 12 g) did not improve the results significantly. Ghrelin, IR cell amounts, and cold carcass weight in contrast to the respective prebiotic and synbiotic group results did not show supportive results.

Due to the ghrelin IR cell amount results, it can be concluded that ghrelin IR cells in 12-week-old calves are significantly more in *the abomasum fundus* region. Medium (6 g/per day) and highest (12 g/per day) prebiotic (inulin) addition to food uptake increases food breakdown by having a longer feeling of satiety and increased calf weight without an increase in food uptake amount. Synbiotic *Enterococcus faecium* (2^*∗*^10^9^ CFU/g) addition to food together with prebiotic inulin does not improve these results significantly.

## Figures and Tables

**Table 1:** Ghrelin, IR, cell number 1 mm^2^, and average cold carcass weight (kg) of 12-week-old calves fed with different amounts of inulin and synbiotic.

Group	Reaction intensity		Cold carcass weight (kg ± SD)
	*Pars pylorica*	*Abomsum fundus*	
CoG	5 ± 4.22	13 ± 8.01	42.6 ± 6.88
PreG6	0 ± 0.07^a^	15 ± 5.86	44.8 ± 0.99^c^
PreG12	0 ± 0.05^a^	6 ± 4.50^a^	51.4 ± 2.76^a^
PreG24	0 ± 0.04^a^	4 ± 3.99^a^	54.0 ± 2.89^a^
SynG6	0 ± 0.89^a^	10 ± 3.27^b^	49.7 ± 2.41^b^
SynG12	0 ± 0.11^a^	5 ± 2.93^a^	52.3 ± 1.61^a^
SynG24	0 ± 0.03^a^	2 ± 0.79^a^	49.6 ± 1.85^b^

Data are presented as mean ± S.D. a- significantly compared to CoG (*p* < 0.01); b- significantly compared to CoG (*p* < 0.05); c- not significantly compared to CoG (*p* > 0.05).

**Table 2:** Chemical composition of concentrated feed and Jerusalem artichokes flour for study animals.

Flour name	Composition (g·kg^−1^ dry matter basis)						Composition (g·mg^−1^ dry matter basis)			
	Dry matter, g kg^−1^	CP	NDF	ADF	Starch	Inulin	Free glucose	Free fructose	Saccharose	Nucleic acids
Concentrated feed	882	142	481	34	655	—	—	—	—-	—
Jerusalem artichoke	948–956	171	—	—	628–645	485–501	8	26	106	21

CP- crude protein; NDF- neutral detergent fiber; and ADF- acid detergent fiber.

## Data Availability

Dornonville de la Cour C, Björkqvist M, Sandvik AK. 2001. A-like cells in the rat stomach contain ghrelin and do not operate under gastrin control.Regul Pept. 99 : 141–150.2. Hamasalim HJ. 2016. Synbiotic as Feed Additives Relating to Animal Health and Performance. Adv Microbiol. 6 : 288–302.3. Hayashida T, Murakami K, Mogi K, Nishihara M, Nakazato M, Mondal MS, Murakami N. 2001. Ghrelin in domestic animals: distribution in the stomach and its possible role. Domest. Anim. Endocrinol. 21 : 17–24.4. Hayashi H, Yamaguchi M, Kozakai T. 2020, Leptin and ghrelin expressions in the gastrointestinal tracts of calves and cows. J Vet Med Sci. 82 : 475–478.5. Özfili N, Tütüncü Ş, Cetin M, Udum D. 2011b. Effects of different feeding programs and ghrelin injection on plasma ghrelin concentrations and distribution of the ghrelin positive cells in the abomasum of Awassi male lambs. Revue Med Vet. 162 : 65–71.
